# Comprehensive evaluation of risk factors for aseptic loosening in cemented total knee arthroplasty: A systematic review and meta‐analysis

**DOI:** 10.1002/jeo2.12095

**Published:** 2024-07-21

**Authors:** Kaiyi Yao, Yao Chen

**Affiliations:** ^1^ Faculty of Medicine and Health Sciences Ghent University Ghent Belgium; ^2^ Department of Applied Mathematics, Computer Science and Statistics Ghent University Ghent Belgium; ^3^ Department of Morphology, Imaging, Orthopedics, Rehabilitation and Nutrition Ghent University Merelbeke Belgium; ^4^ DIGPCR‐Ghent University Digital PCR Consortium Ghent University Merelbeke Belgium

**Keywords:** aseptic loosening, cemented, risk factor, total knee arthroplasty

## Abstract

**Purpose:**

Aseptic loosening is the most common cause for revisions after total knee arthroplasty (TKA). Despite many studies exploring various risk factors associated with aseptic loosening, findings often present inconsistencies. To address this, we conducted a thorough review of the literature to identify and analyse these risk factors in cemented TKA. Additionally, we performed a meta‐analysis to reconcile the divergent conclusions observed across studies.

**Methods:**

We searched PubMed, Web of Science and Embase from 1996 up to 2024 and evaluated the quality of the included literature. Seventy‐four studies were included to assess the association of BMI, diabetes, high physical activity (HPA), osteoporosis, rheumatoid arthritis (RA), cement material and implant design. Twenty‐nine studies were used to calculate relative risk and CIs (using the random effects theory) and study heterogeneity for six different risk factors (BMI, diabetes, HPA level, cement material, polyethylene and implant design).

**Results:**

Patients with diabetes are eight times more likely to experience aseptic loosening compared to those without diabetes (RR = 9.18, 95% CI: 1.80−46.77, *p* < 0.01). The use of tibial stem extension or highly crosslinked polyethylene can help reduce the incidence of aseptic loosening. However, we did not identify BMI, HPA, osteoporosis, RA, the use of high‐viscosity cement and the utilization of mobile‐bearing designs as risk factors for aseptic loosening post‐cemented TKA.

**Conclusions:**

Patients with diabetes undergoing TKA should be counselled regarding their potential increased risk of aseptic loosening. The use of tibial stem extensions and HXLPE can mitigate the incidence of aseptic loosening in cemented TKA. However, given a limited number of studies were included in the meta‐analysis, we believe that higher‐level studies are necessary to clearly identify other risk factors.

**Level of Evidence:**

Level III.

**Abbreviations**
BMI: body mass index
HPA: high physical activity
HVC: high viscosity cement
HXLPE: highly crosslinked polyethylene
LCS: low‐contact stress
LVC: low viscosity cement
NOS: Newcastle−Ottawa scale
OA: osteoarthritis
RA: rheumatoid arthritis
TKA: total knee arthroplasty

## INTRODUCTION

Aseptic loosening, characterized by the gradual separation of the implant from the bone without the presence of infection, is a primary cause of total knee arthroplasty (TKA) failures. Although TKA has low revision rates, the absolute numbers steadily increase. Despite the advancements in surgical techniques and implant design, aseptic loosening remains a persistent concern, leading to revision surgeries, less satisfactory patient outcomes and increased healthcare costs [14]. As such, optimizing primary outcomes in total joint arthroplasties is crucial to mitigate the challenges and complications associated with revisions.

Although the reasons for aseptic loosening are not fully understood, one of the prevailing theories regarding pathophysiology implicates the generation of debris particles on implant surfaces. These particles trigger an inflammatory response that disrupts bone homoeostasis, leading to local osteolysis and, ultimately, aseptic loosening of the prosthesis [48]. Several factors have been proposed which can increase the risk of patients developing aseptic loosening after TKA. Those factors are commonly categorized into host‐, genetic‐, surgical‐ and prosthesis‐related factors. Understanding the specific risk factors contributing to aseptic loosening is crucial for optimizing patient selection, surgical strategies and postoperative care.

This study incorporates various risk factors of aseptic loosening, encompassing host‐related factors such as BMI, diabetes, osteoporosis and rheumatoid arthritis (RA), as well as considerations regarding surgical factors such as the type of cement used and the implant design. In contrast to prior studies that often examined risk factors in isolation or studies which focused only on host factors [17], our research adopts a more extensive approach. Our study specifically investigates cemented TKA as the cemented fixation is still most used in TKA, thanks to extensive clinical experience and favourable clinical outcomes [65]. Additionally, we investigated whether high physical activity (HPA) contributes to the risk of aseptic loosening. There are increased desires and expectations of patients regarding continued participation in sports activities after TKA. Thirty‐four percent of patients who underwent TKA reported engaging in at least one sporting activity at the 5‐year mark postoperation [47]. However, there is considerable debate regarding the amount and type of physical activity that orthopaedic surgeons can confidently advise their patients [40]. Kornuijit et al. conducted a meta‐analysis examining HPA post‐TKA, investigating the association between activity levels and the risk of revision surgery for all causes. Their study also did not distinguish between cemented and cementless [58]. Our study focuses on aseptic loosening as the cause of revision surgery in cemented TKA instead.

Therefore, the primary goal of this systematic review is to comprehensively evaluate the existing literature to identify and analyse the risk factors associated with aseptic loosening in cemented TKA. This study provides an updated review of the current literature by synthesizing findings from recent research while also incorporating insights from previous studies. The secondary goal is to reconcile inconsistent conclusions by aggregating available data through meta‐analysis.

## METHODS

### Search strategy

A systematic literature search in accordance with Preferred Reporting Items for Systematic Reviews and Meta‐Analyses (PRISMA) guidelines was conducted across the following databases: PubMed, Web of Science and Embase. The search terms comprised a combination of medical subject headings (MeSH) and keywords related to aseptic loosening, cemented TKA and risk factors. All types of indexed publications written in English were considered. The search was limited to studies published between 1996 and 2024 to ensure relevance. In this way, there were 4134 publications identified.

### Study selection and quality assessment

**Figure 1:**
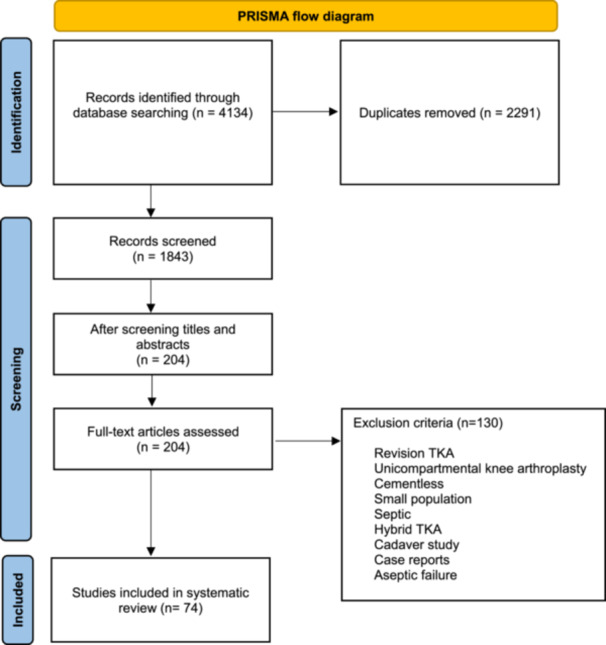
PRISMA 2020 flow diagram for new systematic reviews. PRISMA, Preferred Reporting Items for Systematic Reviews and Meta‐Analyses; TKA, total knee arthroplasty.

The inclusion criteria for the studies outline primary TKA with cemented fixation and aseptic loosening, with publications ranging from 1996 to 2024. The screening of titles and abstracts for eligibility yielded 204 potential publications. Subsequent full‐text review of the potentially relevant articles resulted in the inclusion of 74 publications. The exclusion criteria can be seen in Figure 1.

The quality of the included studies was assessed against the Newcastle−Ottawa Scale (NOS) [93]. Two reviewers were involved in the quality assessment (K. Y. and Y. C.), with any disagreements resolved by consensus and review.

### Data synthesis and analysis

Descriptive statistics were used to summarize study characteristics, while tables and figures were employed to present the data. Interrater reliability for all dual‐screened processes was assessed by calculating the proportional agreement between assessors. The risk of aseptic loosening was reported as a dichotomous outcome. In the initial stage of the meta‐analysis, when the outcome was dichotomous, the number of events and total number of participants were extracted. Effect sizes in the form of relative risk with their 95% CIs were then calculated for each study, which were presented by risk factors. To handle heterogeneity among studies, restricted maximum likelihood random effects estimation was used. The Mantel−Haenszel method was employed since this method has been shown to have better statistical properties when there are few events [42]. Additionally, statistical heterogeneity was assessed by means of an $I^{2}$ test and was categorized as low (<50%), moderate (51%–75%) or high according to predefined criteria [43]. Influence analysis using the leave‐one‐out method was also conducted to identify outliers and influential cases that could impact the validity and robustness of the meta‐analysis conclusions. Egger's test was only conducted for the implant design as less than 10 studies were pooled for other risk factors. The level of statistical significance was set at *p* < 0.05 for all tests. All analyses were conducted using R [80] (version 4.2.2).

## RESULTS

### Study characteristics

Study characteristics are reported in Table 1. The systematic review included cases, with a median sample size across studies of 236 (range: 13–418,054). Sixty‐five percent of participants were women. All studies were conducted in adults (mean age = 66.3). Studies were conducted in Australia (*n* = 1), Austria (*n* = 2), Canada (*n* = 2), China (*n* = 1), Egypt (*n* = 1), France (*n* = 3), Germany (*n* = 2), India (*n* = 1), Iran (*n* = 1), Israel (*n* = 1), Italy (*n* = 2), Japan (*n* = 2), Netherlands (*n* = 4), New Zealand (*n* = 1), Norway (*n* = 2), Slovakia (*n* = 1), Spain (*n* = 1), South Korea (*n* = 7), Thailand (*n* = 1), Turkey (*n* = 2), UK (*n* = 2) and the USA (*n* = 35). Studies were investigating the association between aseptic loosening after cemented TKA and BMI (*n* = 9), diabetes (*n* = 4), osteoporosis (*n* = 1), RA (*n* = 3), HPA (*n* = 4), cement type (*n* = 6), implant design (*n* = 34), polyethylene (*n* = 5) and short‐stemmed tibial component (*n* = 8).

**Table 1:** Overview of the included studies.

Study	Publication year	Country	Total cases	Mean age (years)	Sex (female %)	Mean follow‐up (years)	Study design	Quality assessment	Mean BMI (kg/m²)
BMI									
Abdel et al. [1]	2015	USA	5088	69	60	7 (range, 2–15)	Retrospective cohort study	7	33
Başdelioğlu [5]	2021	Turkey	588	67.25	56	4.34	Retrospective cohort study	7	/
Garceau et al. [26]	2020	USA	236	65.2	60	3.0	Retrospective cohort study	7	32.2
Lim et al. [64]	2017	USA	160	61.4	54	range 5–10	Retrospective cohort study	8	31.7
Crawford et al. [19]	2017	USA	1851	62.2	69	5.4 (range 2–9.4)	Observational registry study	/	41.7
Hakim et al. [34]	2019	Israel	374	64.3	68	10.8	Retrospective cohort study	8	/
Winiarsky et al. [94]	1998	USA	1818	67.6	66	4.8	Retrospective cohort study	8	/
Griffin et al. [33]	1998	USA	73	67.8	73	10.6	Retrospective cohort study	8	/
Krushell et al. [60]	2007	USA	78	68.15	/	7.5	Retrospective cohort study	8	34.85
Diabetes									
Meding et al. [69]	2003	USA	5220	70	41	4.33	Retrospective cohort study	7	/
Deng et al. [20]	2023	Australia	440	64.4	56	7	Retrospective case‐control study	6	27.2
Maradit Kremers et al. [59]	2017	USA	16,085	66.2	54	6.1	Retrospective cohort study	8	/
Papegelopoulos et al. [72]	1996	USA	1634	70	51	8	Retrospective cohort study	7	/
Osteoporosis									
Harris et al. [38]	2023	USA	418,054	64.4	62	5	Retrospective cohort study	9	/
RA									
Böhler et al. [8]	2018	Austria	137	/	/	/	Retrospective registry study	7	/
Schreiner et al. [85]	2023	Austria	251	59	78	5.17	Retrospective cohort study	7	/
Feng et al. [22]	2013	China	297	61.47	83	10	Retrospective study	9	24.7
HPA									
Crawford et al. [18]	2020	USA	3530	63.8	65	11.4	Retrospective observational study	7	33.9
Mont et al. [71]	2007	USA	114	70	61	7	Retrospective cohort study	7	29
Ponzio et al. [76]	2018	USA	2016	66.3	43	Up to 10	Retrospective cohort study	7	28.3
Ennis et al. [21]	2024	USA	298	63.5	46	8.2	Retrospective cohort study	8	27.5
Cement									
Wyatt et al. [97]	2021	USA	76,052	68.02	62	4.4	Retrospective cohort study	7	31.1
Buller et al. [12]	2020	USA	10,014	66.6	63	2.8	Retrospective cohort study	8	30.2
Foran et al. [23]	2011	USA	529	61	/	1.42	Case series	/	/
Kopinski et al. [57]	2016	USA	13	62.4	54	/	Case series	/	32.1
Crawford et al. [19]	2017	USA	1851	62.2	69	5.4 (range 2–9.4)	Observational registry study	/	41.7
Arsoy et al. [3]	2013	USA	1337	58	/	1.4	Retrospective case‐control study	7	35.6
Mobile bearing versus fixed bearing									
Gøthesen et al. [31].	2013	Norway	17,772	70.2	78	1.8–6.9	Retrospective cohort study	9	/
Song et al. [88]	2020	South Korea	200	68.5	97	4.95	Retrospective cohort study	7	26.4
Lacko et al. [63]	2019	Slovakia	1543	69.7	70	8.3	Prospective cohort study	7	31.1
Kim et al. [55]	2007	South Korea	292	69.8	95	13.2	Retrospective cohort study	8	27.5
Bistolfi et al. [7]	2013	Italy	172	70	81	9.67	Prospective cohort study	7	/
Powell et al. [78]	2017	New Zealand	190	65.5	34	10	Randomized controlled trial	7	29.7
Kalisvaart et al. [49]	2012	USA	152	67.3	70	5	Randomized controlled trial	8	32.0
Shemshaki et al. [87]	2012	Iran	300	69	64	5	Randomized controlled trial	8	/
Woolson et al. [96]	2011	USA	63	78	/	11.5	Randomized controlled trial	7	28.4
Rahman et al. [82]	2010	Canada	51	62.3	62.7	3.5	Randomized controlled trial	7	31.4
Hanusch et al. [36]	2010	UK	105	69.7	49.5	1.1	Randomized controlled trial	7	29.8
Matsuda et al. [68]	2010	Japan	61	74.5	77	5.7	Randomized controlled trial	8	/
Gioe et al. [28]	2009	USA	312	72.2	2.8	3.5	Randomized controlled trial	7	31.8
Wohlrab et al. [95]	2009	Germany	60	65.5	56.7	5	Randomized controlled trial	7	24.3
Harrington et al. [37]	2009	USA	140	63.5	64.3	2	Randomized controlled trial	8	34.2
Hasegawa et al. [39]	2009	Japan	50	72	88	3.3	Randomized controlled trial	8	/
Kim et al. [51]	2001	South Korea	240	65	69	7.4	Randomized controlled trial	7	/
Kim et al. [54]	2019	South Korea	328	63	86.5	17	Randomized controlled trial	7	28
Kim et al. [53]	2018	South Korea	184	61.5	81.5	12	Randomized controlled trial	7	26.2
Van Hamersveld et al. [92]	2018	Netherlands	46	67.5	76.1	6	Randomized controlled trial	6	30
Chaudhry et al. [15]	2018	India	110	58.1	54.5	6−8	Randomized controlled trial	6	25.4
Abdel et al. [2]	2018	USA	169	67.1	65.6	10	Randomized controlled trial	8	/
Baktir et al. [4]	2016	Turkey	93	64.8	88.2	8	Randomized controlled trial	7	/
Fransen et al. [25]	2015	Netherlands	237	65.8	69.6	5	Randomized controlled trial	7	30.2
Breugem et al. [11]	2014	Netherlands	69	79.2	65.2	7.9	Randomized controlled trial	6	/
Breeman et al. [10]	2013	UK	539	69	60.1	5	Randomized controlled trial	8	30
Prasad et al. [79]	2013	India	32	63.7	62.5	1	Randomized controlled trial	6	/
Radetzki et al. [81]	2013	Germany	39	66	10.8	10.8	Randomized controlled trial	7	29.5
Kim et al. [50]	2012	South Korea	216	45	76.9	16.8	Randomized controlled trial	7	/
Scuderi et al. [86]	2012	USA & Canada	293	63.5	58.4	4	Randomized controlled trial	7	29.5
Pijls et al. [75]	2012	Netherlands	42	65	81	10−12	Randomized controlled trial	7	27
Mahonney et al. [67]	2012	USA	507	66	63.9	2	Randomized controlled trial	8	31
Lizaur‐Ultrilla et al. [66]	2012	Spain	119	74.3	79	2	Randomized controlled trial	8	31.9
Tienbon et al. [91]	2012	Thailand	200	69.2	85.5	2	Randomized controlled trial	7	26.3
Short‐stemmed tibial component									
Garceau et al. [27]	2022	USA	1350	/	/	4.4	Retrospective cohort study	7	/
Garceau et al. [26]	2020	USA	236	65.2	60	3.0	Retrospective cohort study	7	32.2
Park et al. [73]	2018	South Korea	602	66.9	96	8.71	Retrospective cohort study	8	27.4
Hinman et al. [44]	2021	USA	111,937	66.6	61	2.5	Retrospective cohort study	8	31.1
Fournier et al. [24]	2020	France	140	69.45	21	4.21	Retrospective cohort study	7	34.6
Steere et al. [89]	2018	USA	178	61.72	81.5	2.74	Retrospective cohort study	8	41.1
Parratte et al. [74]	2017	France	120	68.25	17.5	3	Randomized controlled trial	7	35.5
Mohammad et al. [70]	2023	Egypt	264	57	87	6.1	Randomized controlled trial	7	35.4
Polyethylene									
Kindsfater et al. [56]	2015	USA	926	66.3	77.9	Minimum 5	Randomized controlled trial	8	32.9
Lachiewicz et al. [62]	2016	USA	232	69	61.3	4.5	Randomized controlled trial	8	31
Boyer et al. [9]	2018	France	27,013	71	88.8	5.9	Retrospective observational study	7	/
Hodrick et al. [45]	2008	USA	200	68.5	59	6.9	Retrospective cohort study	8	/
Giustra et al. [29]	2023	Italy	128	71.5	75	12.5	Retrospective cohort study	7	/

Abbreviation: HPA, high physical activity.

### Quality assessment

Of the 74 eligible studies, 36 were retrospective studies, 35 were prospective studies, two were case series and one was an observational study. Of 36 retrospective studies, 34 were cohort studies and two case‐control studies. According to the NOS scoring system, none of the cohort studies or case‐control studies was of poor quality (≤1 point). One retrospective case‐control study and four randomized controlled trials were of fair quality (2−6 points), and other studies were considered good quality (≥7 points) [93].

### Risk of aseptic loosening

#### BMI

Of the nine studies examined, three reported a positive association between a higher BMI and the risk of aseptic loosening [1, 5, 26]. Abdel et al. observed that individuals with a BMI ≥ 35 kg/m² had a higher likelihood of revision TKA due to aseptic tibial loosening compared to those with a BMI < 35 kg/m², with data reported at two‐time points: 5 and 15 years [1]. Başdelioğlu observed a trend of increasing incidence of aseptic loosening with higher BMI categories, ranging from absence in patients with BMI < 30 kg/m² to a peak of 4.7% in those with BMI > 40 kg/m² [5]. Garceau et al. reported a higher rate of aseptic loosening in individuals with a BMI > 40 kg/m² [26]. Lim et al. suggest that maintaining stable weight following primary TKA is linked to a decreased risk of late revisions (>10 years) attributed to aseptic loosening [64]. In contrast, Crawford et al. only found one instance of aseptic tibial loosening in the obese group [19]. Several other studies did not identify a significant association between a high BMI and aseptic loosening either [33, 34, 60, 94].

#### Diabetes

In the four studies reviewed, Meding et al. and Deng et al. reported a positive association between diabetes and aseptic loosening [20, 69]. Meding et al. observed a statistically higher rate of aseptic loosening in patients with diabetes compared to those without (3.6% vs. 0.4%) [69], while Deng et al. found significantly higher odds of diabetes in the aseptic loosening group compared to controls (OR = 2.78, *p* = 0.01) [20]. Papegelopoulos et al. found a higher rate of aseptic loosening for primary TKA (7.4%) in patients with diabetes, although the difference is not significant compared to patients without diabetes [72]. Conversely, Kremers et al. did not observe a significant difference in the risk of aseptic loosening between diabetics and nondiabetics (HR = 0.87) but did identify an association between presurgery hyperglycaemia and increased risk of aseptic loosening (HR = 4.95) [59].

#### HPA

All studies we investigated related to HPA exhibited no significant association between the HPA level and aseptic loosening [18, 21, 71]. Although Ponzio et al.'s study indicates an eightfold higher risk of aseptic loosening for the HPA group (HPA group: 0.8%; LPA group: 0.1%), the difference did not reach significance [76].

#### Osteoporosis

Limited research exists regarding the outcomes of TKA in patients with osteoporosis. Harris et al. discovered that individuals with a history of osteoporosis faced a 20% higher risk of aseptic loosening within 5 years compared to those without a history of osteoporosis (HR: 1.2; 95% CI: 1.1−1.3; *p* < 0.001) [38].

#### RA

The three studies we reviewed investigating the risk of aseptic loosening in patients with RA all identified a positive association. Böhler et al. highlighted that elevated inflammatory disease activity heightens the risk of radiographic loosening following TKA in patients with RA (RA: 34.4%; osteoarthritis [OA]: 6.5%; *p* = 0.001) [8]. Their study also revealed a protective effect of biological DMARDs against the risk of radiographic component loosening, which is supported by Schreiner et al. [85] Feng et al. observed that patients with OA had higher survival rates for prostheses compared to those with RA, with 10‐ and 15‐year survival rates of 93.6% ± 1.8% and 92.7% ± 2%, respectively, for OA patients, and 88% ± 5% and 78.3% ± 7.9%, respectively, for RA patients. It's important to note that these results reflect overall prosthesis survival rates and do not specifically address aseptic loosening [22].

#### Cement material

Three studies have suggested a potential association between high‐viscosity cement (HVC) and early aseptic loosening following TKA [23, 57]. Foran et al. reported eight patients who received HVC and experienced early aseptic loosening [23]. Kopinski et al. reported 13 cases of tibial component debonding, with all patients having undergone TKA using HVC [57]. In a study by Buller et al., it was found that the rate of revision for aseptic loosening was significantly higher in the HVC cohort (1.9%) compared to the low‐viscosity cement (LVC) cohort (0.92%) (*p* < 0.001) [12].

Three other studies did not find an association between the use of HVC and aseptic loosening [3, 19, 97]. Crawford et al. did not find an association between the use of HVC and aseptic loosening and concluded that HVC can be used in most patients, including the high‐risk obese group, with low rates of tibial aseptic loosening [19]. Arsoy et al. reported a 1.9% aseptic loosening rate using LVC, which is comparable to the rate of aseptic loosening in HVC groups of other studies [3]. Wyatt et al. also did not observe a significant association between the use of HVC and aseptic loosening [97].

#### Mobile bearing versus fixed bearing

The 34 studies investigating mobile bearing have yielded varying findings regarding its association with the risk of aseptic loosening. Gøthesen et al. reported a sixfold higher risk of aseptic loosening with the low‐contact stress (LCS) mobile‐bearing implant in primary arthroplasty in Norway compared to the best‐performing design (for the LCS classic, RR: 6.8; 95% CI: 3.8−12.1) [31]. Song et al. also found a sixfold higher incidence of revision TKA due to aseptic loosening in a mobile‐bearing group compared to a fixed‐bearing group (7% vs. 1%, *p* = 0.032) [88]. On the contrary, Lacko et al. reported contrasting findings, suggesting that mobile‐bearing implants were linked to a significantly reduced risk of total (RR = 0.46; *p* = 0.049) and late revisions due to aseptic loosening (RR = 0.14; *p* = 0.008) [63]. Twenty other studies observed no significant difference between the two groups [2, 7, 10, 11, 25, 28, 49, 50, 53, 54, 55, 66, 67, 75, 78, 81, 86, 91, 92, 96]. Eleven studies did not observe aseptic loosening in either group [4, 15, 36, 37, 39, 51, 68, 79, 82, 87, 95].

#### Short‐stemmed tibial component

Studies related to short‐stemmed tibial components observed a higher rate of aseptic loosening [24, 27, 74], and the use of tibial stem extensions for short‐stemmed tibial components may decrease the risk of aseptic loosening [26, 44, 73]. Hinman et al. conducted a sizable cohort study involving 10,476 individuals who underwent cemented TKA. Their findings revealed a reduced risk of revision attributed to aseptic loosening among patients who received a tibial stem (HR: 0.38; 95% CI: 0.17−0.85) [44]. Garceau et al. observed that short, native tibial stem design is associated with early aseptic loosening in primary cemented TKA through a multicenter cohort study. The authors observed that the overall survival rate at 5 years was superior for the short tibial stem extension cohort compared to the nonstemmed group (overall survival: 100% vs. 94.5%, *p* = 0.006) [26]. Park et al. found that the overall implant survival rate was significantly higher in the stem group than in the nonstemmed group (*p* = 0.0201) [73]. Two studies did not observe aseptic loosening in either group [70, 89].

#### Polyethylene

Five studies showed a higher rate of aseptic loosening in the conventional polyethylene group compared to the HXLPE group, but none reached statistical significance [56].

Conversely, two studies found no cases of aseptic loosening in either group [52, 90], and Giustra et al. did not report a significantly higher rate in either group [29].

### Meta‐analysis

**Figure 2:**
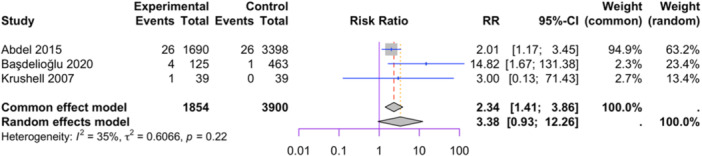
Comparison of relative risk of aseptic loosening between BMI > 35 kg/m^2^ and BMI < 35 kg/m^2^: forest plot of effect sizes.

**Figure 3:**
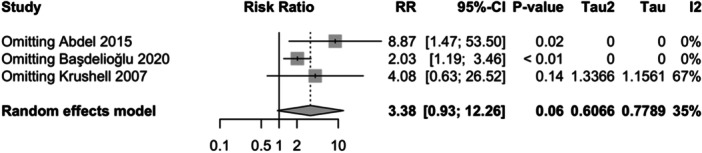
Relative risk, CI, tau and $I^{2}$ (BMI): influence analysis plot of effect sizes.

Three studies reporting BMI were included in the meta‐analysis (1854 in group BMI > 35 kg/m^2^ $m^{2}$ and 3900 in group <35 kg/m^2^, see Figure 2), as they included control groups, allowing for the relative risk of aseptic loosening to be assessed within each category [1, 5, 60]. The random‐effect model did not show a significant difference in relative risk between the two groups (RR = 3.38, 95% CI: 0.93−12.26, *p* = 0.0635). Low heterogeneity was observed among the studies ($I^{2}$ = 35%, *p* = 0.22). Influence analysis results indicate that the exclusion of Abdel et al. and Başdelioğlu results in a considerable alteration in the relative risk estimates (more than 20% increase), whereas the removal of Krushell et al. leads to relatively minor changes (see Figure 3).

**Figure 4:**
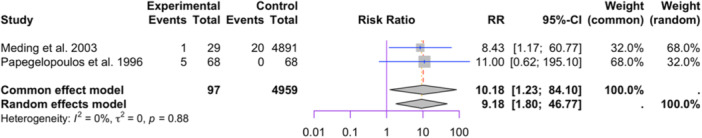
Comparison of relative risk of aseptic loosening between patients with or without diabetes: forest plot of effect sizes.

Two studies reporting diabetes were included in the meta‐analysis (97 in the diabetes group and 4959 in the nondiabetes group, see Figure 4) [69, 72]. The random‐effect model shows a significant result (RR = 9.18, 95% CI: 1.80−46.77, *p* < 0.01). There was no evidence of statistical heterogeneity, as $I^{2}$ = 0.

**Figure 5:**
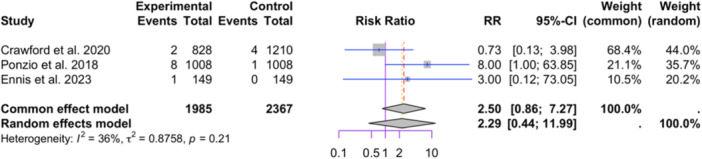
Comparison of relative risk of aseptic loosening between patients with high or low physical activity: forest plot of effect sizes.

**Figure 6:**
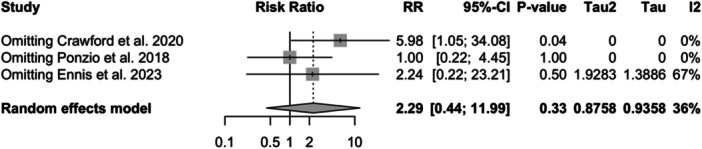
Relative risk, CI, tau and $I^{2}$ (HPA): influence analysis plot of effect sizes. HPA, high physical activity.

Three studies reporting physical activity were included in the meta‐analysis (1985 in the HPA group and 2367 in the low physical activity group; see Figure 5) [18, 21, 76]. The random‐effect model did not show a significant difference in the relative risk between the two groups (RR = 2.29, 95% CI: 0.44−11.99, *p* = 0.3275). Low heterogeneity was observed among the studies ($I^{2}$ = 36%, *p* = 0.21). Influence analysis results indicate that omitting Crawford et al. or Ponzio et al. leads to a large change in the relative risk (see Figure 6). With the removal of Crawford et al., the difference in relative risk between the two groups will be significant (RR = 5.98, 95% CI: 1.05−34.08, *p* = 0.0442).

**Figure 7:**
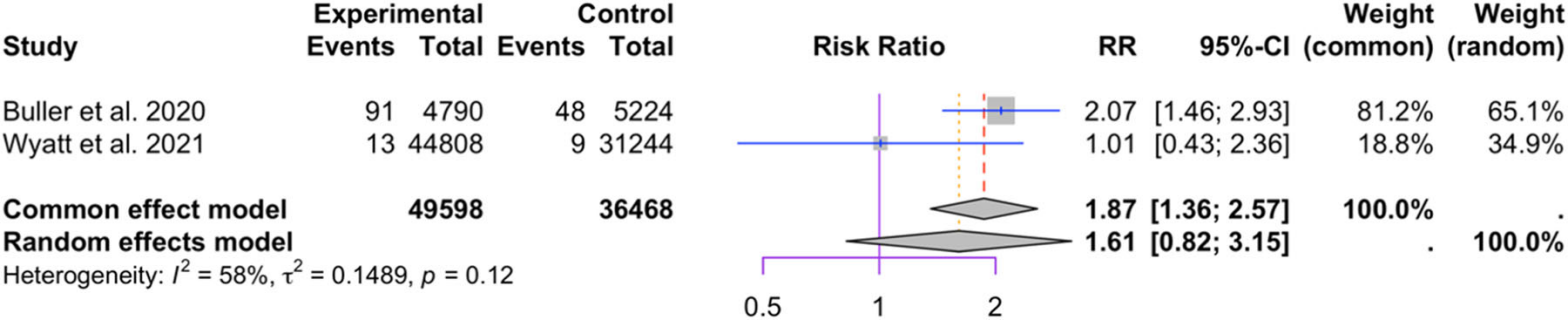
Comparison of relative risk of aseptic loosening between patients receiving HVC or LVC: forest plot of effect sizes. HVC, high‐viscosity cement; LVC, low‐viscosity cement.

Two studies reporting cement material were included in the meta‐analysis (49,598 in the HVC group and 26,468 in the LVC group, see Figure 7) [12, 97]. The random‐effect model shows no significant difference (RR = 1.61, 95% CI: 0.82−3.15, *p* = 0.1652). Moderate heterogeneity was observed among the studies ($I^{2}$ = 58%, *p* = 0.12).

**Figure 8:**
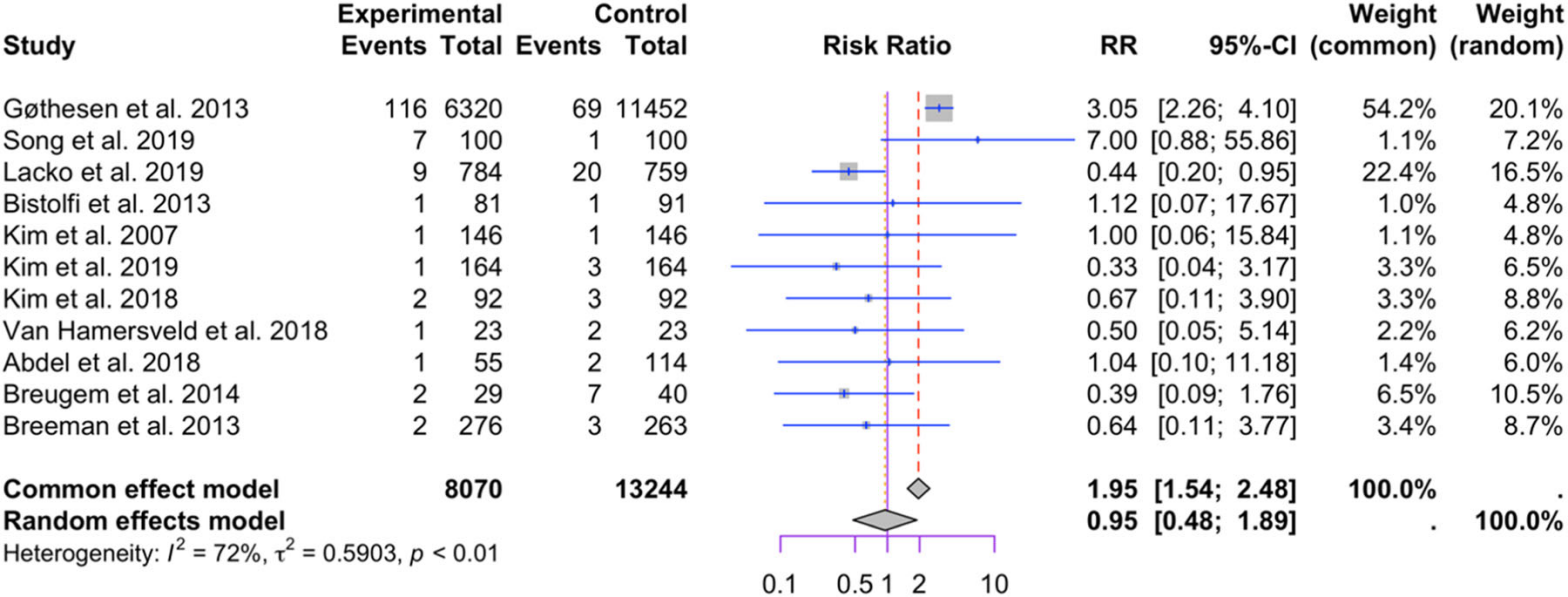
Comparison of relative risk of aseptic loosening between patients with mobile bearing or fixed bearing: forest plot of effect sizes.

**Figure 9:**
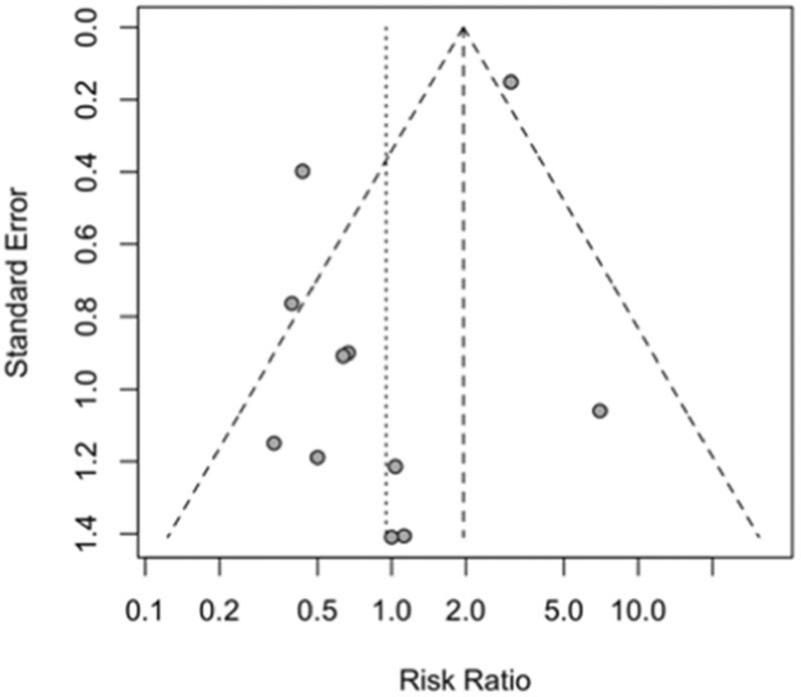
Funnel plot of studies on implant design (mobile‐bearing vs. fixed‐bearing).

Eleven studies reporting implant designs were included in the meta‐analysis (8070 in the mobile bearing group and 13,244 in the fixed bearing group, see Figure 8) [2, 7, 10, 11, 31, 53, 54, 55, 63, 88, 92]. The random‐effect model did not show a significant difference between the two groups (RR = 0.95, 95% CI: 0.48−1.89, *p* = 0.6732). High heterogeneity was observed among the studies ($I^{2}$ = 72%, *p* < 0.01). Egger's test suggested potential publication bias (t = −2.39, *df* = 9, *p* = 0.0404) and the funnel plot exhibited asymmetry (see Figure 9). Note that, we initially attempted to include studies with zero events in one group in this meta‐analysis. However, the algorithm failed to converge with their inclusion. Despite the exclusion of studies with zero events in one group, we still retained eleven studies for data pooling in this meta‐analysis. Thus, we decided to remove those studies from the meta‐analysis [25, 28, 49, 50, 66, 67, 75, 78, 81, 86, 91, 96].

**Figure 10:**
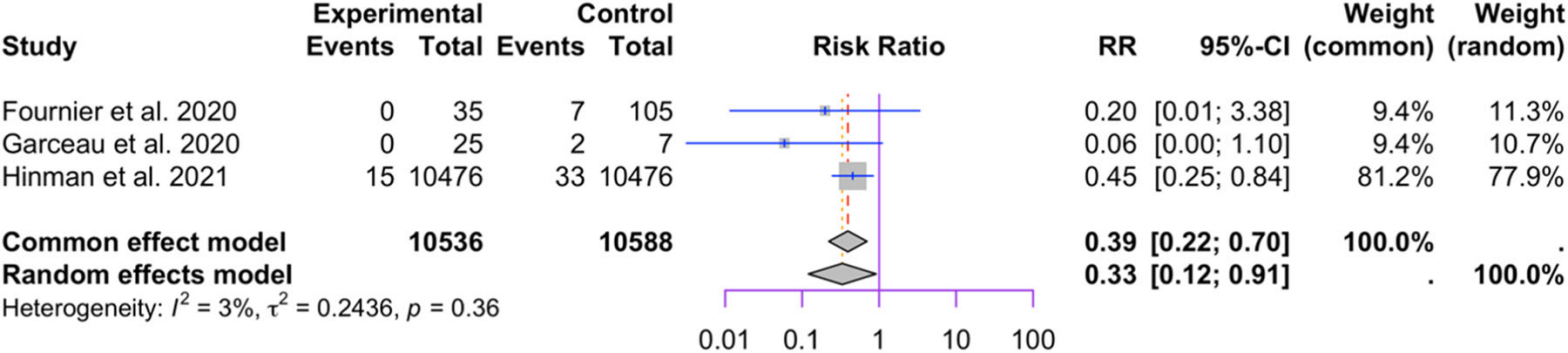
Comparison of relative risk of aseptic loosening between patients with stemmed or nonstemmed tibial implants: forest plot of effect sizes.

**Figure 11:**
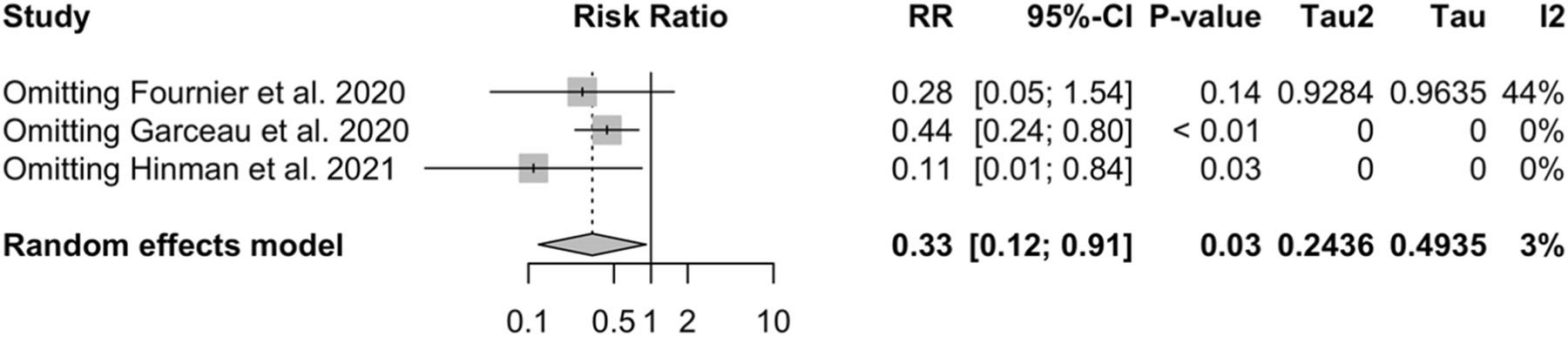
Relative risk, CI, tau and $I^{2}$ (stemmed tibial implant): influence analysis plot of effect sizes.

Three studies reporting stemmed tibial components were included in the meta‐analysis (10,536 in stemmed tibial components and 10,588 in the nonstemmed tibial components group, see Figure 10) [24, 26, 44] The random‐effect model showed a significant difference between the two groups (RR = 0.33, 95% CI: 0.12−0.91, *p* = 0.0324). Low heterogeneity was observed among the studies ($I^{2}$ = 3%, *p* = 0.36). Influence analysis results show exclusion of Fournier et al. leads to insignificant differences in the two groups (see Figure 11).

**Figure 12:**
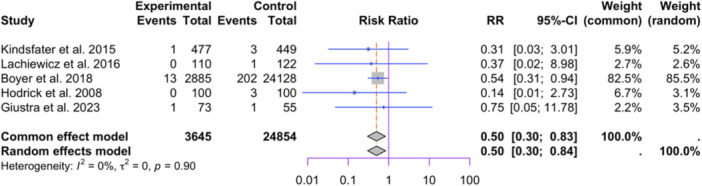
Comparison of relative risk of aseptic loosening between patients with HXLPE or conventional polyethylene: forest plot of effect sizes.

**Figure 13:**
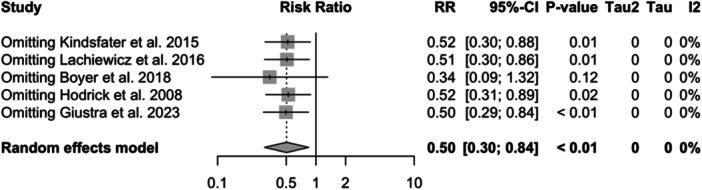
Relative risk, CI, tau and $I^{2}$ (polyethylene): influence analysis plot of effect sizes.

Five studies reporting polyethylene types were included in the meta‐analysis (3645 in HXLPE and 24,854 in the conventional polyethylene group; see Figure 12 [9, 29, 45, 56, 62]. The random‐effect model shows a significant between the two groups (RR = 0.5, 95% CI: 0.30−0.84, *p* = 0.0093). Low heterogeneity is observed among the studies ($I^{2}$ = 0%, *p* = 0.90). Influence analysis results show that the exclusion of the large registry study by Boyer et al. leads to an insignificant difference in the two groups (see Figure 13).

## DISCUSSION

The most significant finding of this study was that patients with diabetes are eight times more likely to experience aseptic loosening compared to those without diabetes. Additionally, our findings suggest that the use of tibial stem extensions and HXLPE can mitigate the incidence of aseptic loosening in cemented TKA. However, it did not identify BMI, HPA, osteoporosis, RA, the use of HVC and the utilization of mobile‐bearing designs as risk factors for aseptic loosening post‐TKA.

### Host factors

In obese patients, the risk of developing OA of the knee increases by 9%–13% per weight added to body mass. This rate increases up to 35% with each 5 kg of weight gain leading to a growing number of obese individuals undergoing TKA [77]. Patients with a BMI ≥ 35 kg/m^2^ were reported to be nearly twice as likely to develop aseptic tibial loosening [1]. However, our meta‐analysis did not yield a significant association. Several studies have also failed to establish a link between increasing BMI and aseptic loosening rates [17, 19]. In a systematic review by Cherian et al., no association between high BMI and aseptic loosening was found [17]. This might be attributed to the small number of events in the included studies, which leads to wide confidence intervals and imprecise estimates of effect size. Additionally, sparse data may limit the power of statistical tests to detect significant differences between groups. Moreover, various studies investigating obesity employed diverse stratification methods, resulting in sparse cases of aseptic loosening within the morbid obesity group. Consequently, we were unable to examine any potential association between morbid obesity and aseptic loosening following TKA, despite several studies having reported a higher rate of aseptic loosening in individuals with a BMI > 40 kg/m² [5, 26].

Diabetes, a complex metabolic disorder, has significant systemic consequences as an inflammatory condition. It is associated with various cytokines, including TNF‐α and IL‐6, which are linked to diabetes development. These cytokines are also associated with the most common proposed pathophysiology of aseptic loosening [20]. Diabetes also negatively impacts bone health, leading to lower bone mineral density in part due to increased osteoclast activity and inhibited osteoblasts [84]. Our study revealed a significantly elevated incidence of aseptic loosening in patients with diabetes [69]. While Papagelopoulos et al. did not observe a statistically significant increase in the rate of aseptic loosening among patients with diabetes after primary TKA, this finding may be attributed to the limited sample size of their cohort study [72]. Upon pooling data from both studies in the meta‐analysis, the outcome indicated an approximately eightfold higher rate of aseptic loosening in patients with diabetes.

With the increasing number of young undergoing TKA, there is also an increasing number of patients performing HPA after TKA. Intense physical activity has the potential to contribute to increased wear, triggering heightened foreign body responses that may lead to aseptic loosening [47]. There are limited studies in the literature examining the link between high or low physical activity and the risk of aseptic loosening. Of the few studies reporting on this link, none reported a significant association [18, 21, 71, 76]. Our meta‐analysis revealed no significant difference in the relative risk of aseptic loosening between the high and low physical activity groups. This aligns with the findings of Kornujit et al., which indicated no elevated risk of revision surgery for all causes in the HPA group [58].

To our knowledge, there are no present large studies about osteoporosis as a risk factor for aseptic loosening, although the association is speculated in References. [6, 16]. A retrospective cohort analysis by Harris et al. observed a higher risk of aseptic loosening in patients with osteoporosis [38]. Further research is warranted to investigate osteoporosis as a potential risk factor for aseptic loosening. Similarly, to this day, there is very limited research carried out about RA as a potential risk factor for aseptic loosening. The systematic inflammation seen in patients with RA may enhance the local inflammation. A study conducted by Böhler et al. reported that elevated inflammatory RA activity leads to a higher risk of radiographic loosening [8]. Together with another study, it seems that RA patients under treatment with biological DMARDS have a reduced risk of radiographic loosening in comparison to RA patients without this treatment [8, 85]. This also suggests that RA's systematic inflammation may lead to aseptic loosening.

### Surgical factors

HVC is often used for its benefits like shorter mixing and waiting phases during polymerization, and longer working and hardening phases. There are multiple studies implying the association between the use of HVC and a higher risk of aseptic loosening [12, 23, 57]. It is suggested that the reason may be a decreased intrusion depth in the cancellous bone with HVC compared to LVC. Besides the theory of decreased intrusion depth, it is also suggested that the stronger exothermic reaction of HVC may cause thermal damage to the bone leading to aseptic necrosis, followed by micromotion and eventually aseptic loosening [12]. However, there are several studies that did not find an association between HVC and aseptic loosening [3, 19]. This agrees with our meta‐analysis result. Another possible factor would be the cementing technique, which is highly surgeon‐dependent and could also be a risk factor.

The mobile‐bearing design TKA was introduced with the aim of reducing shear and tear forces, consequently minimizing insert wear. Furthermore, a mobile‐bearing design is engineered to exhibit less rigidity, resembling the mechanical characteristics of a natural knee. Enhanced patellar tracking has been asserted as one of its advantages [32]. Despite these claims, only a few independent investigators have demonstrated improved functionality with this design [46, 83]. Multiple extensive studies have associated mobile‐bearing design TKAs to an elevated risk of aseptic loosening [31, 32, 61]. Increased aseptic loosening and no clear improved functionality make the use of mobile‐bearing TKAs questionable. Our meta‐analysis suggests no significant difference between the mobile‐bearing and fixed‐bearing groups in terms of relative risk. Our findings are consistent with those of Hantouly et al. [35] Their meta‐analysis, based on data from 50 randomized controlled trials, similarly concluded that there was no significant difference in aseptic loosening across short‐, mid‐ and long‐term follow‐up intervals. Notably, their analysis encompassed studies on both uncemented and cemented TKA, as well as cases where no instances of aseptic loosening were observed in either mobile‐bearing or fixed‐bearing groups. Note that including studies with zero events in both groups may lead to unreliable estimates. Besides, a high level of heterogeneity was observed among the studies of our meta‐analysis since the conclusions are quite mixed. Further research is warranted to potentially identify the use of mobile‐bearing implant design as a risk factor for aseptic loosening.

Several studies propose that incorporating stem extensions for short‐stemmed tibial components may mitigate the risk of aseptic loosening [26, 41, 44, 73]. This proposition finds support in a study that observed heightened rates of tibial aseptic loosening after TKA featuring a short native tibial stem design [27]. The authors of these studies recommend considering stem extensions, particularly in higher‐risk patients such as those who are morbidly obese or have severe preoperatively varus deformity. They also suggest a potential redesign of native short‐stemmed tibial components [26, 27, 73]. Zhou et al. conducted a recent meta‐analysis exploring the effectiveness of tibial stem extensions in mitigating the risk of aseptic loosening among obese patients. The meta‐analysis, comprising seven studies, indicated that stemmed tibial components potentially decreased the likelihood of revision due to aseptic loosening in obese individuals who may experience increased stress at the tibial component (RR = 0.25; 95% CI: 0.07−0.92) [98]. Their study thus focused on obese patients and did not distinguish between cemented and cementless. Our meta‐analysis also found a significantly lower relative risk in the stemmed tibial components group. Notably, there is a lack of published studies advocating for the routine use of tibial stem extensions in primary TKA, primarily due to considerations of cost‐effectiveness.

The adoption of HXLPE in TKA has seen a notable decrease in the reported incidence of failure due to wear over time [13]. Our meta‐analysis further confirms this claim, revealing a reduced incidence of aseptic loosening in the HXLPE group. This finding is consistent with the results of a study by Gkiatas et al., who investigated the impact of HXLPE on TKA revision rates. Their analysis, encompassing over 900,000 cases of all revision causes and over 400,000 specifically for aseptic loosening, compared the outcomes of HXLPE and conventional polyethylene. While the overall revision rates were similar between the two groups (OR = 0.67, 95% CI: 0.39−1.18), a notable difference emerged when examining cases of aseptic loosening alone. In this context, the HXLPE group exhibited a significantly lower revision rate (OR = 0.35, 95% CI: 0.31−0.39) [30]. It is noteworthy that their study included cases of cementless TKA, yet their findings align with our meta‐analysis.

### Strengths and limitations

The outcomes of TKA are affected by a variety of patient, implant and surgical factors. The current investigation represents a comprehensive review of patient and surgical factors affecting TKA due to aseptic loosening. A complex interaction of patient and surgical factors can affect outcomes. Identification of patient factors known to be associated with aseptic failure of TKA allows surgeons to discuss those risks with patients who are under consideration for TKA before surgery. Recognition of surgical factors associated with TKA failures can help surgeons with their choices of surgical techniques and implants.

This review has several limitations. Due to the lack of literature, an analysis of several potential risk factors for aseptic loosenings, such as the cementing technique, young age, use of an intraoperatively tourniquet, thin cement mantle thickness and misalignment, was not performed. The lack of research on these topics suggests potential for future investigation.

Our findings should be interpreted with caution. Due to several factors, such as lack of control groups, absence of raw data, and diverse stratification methods, the meta‐analysis includes a limited number of studies for each risk factor. Consequently, Egger's test to assess publication bias could only be conducted for mobile‐ or fixed‐bearing. Publication bias may lead to an overrepresentation of positive findings. While statistical heterogeneity was low for BMI, diabetes, HPA and cement material, there may still exist clinical and methodological variations among the studies. These differences arise from discrepancies in the age and sex of study populations and the quality of evidence provided by each study. Meta‐regression could not be performed to adjust for these differences due to the insufficient number of studies in the meta‐analysis. Additionally, a high level of heterogeneity was observed for implant designs. Although a random effects model was employed to account for this, elimination of heterogeneity may not be feasible.

## CONCLUSION

In summary, our review underscores the importance of diabetes, tibial stem extensions and polyethylene as significant risk factors for aseptic loosening in cemented TKA. Further research is necessary to fully identify these potential risk factors. Understanding the risk factors for aseptic loosening and implementing preventive strategies are crucial steps in mitigating this undesirable outcome. By doing so, we can potentially reduce the need for TKA revisions, thereby minimizing financial burdens and improving long‐term patient outcomes and satisfaction.

## AUTHOR CONTRIBUTIONS

*Conceptualization*: Kaiyi Yao and Yao Chen. *Data collection*: Kaiyi Yao. *Data analysis*: Yao Chen. *Interpretation of data*: Kaiyi Yao and Yao Chen. All authors read and approved the final manuscript.

## CONFLICT OF INTEREST STATEMENT

The authors declare no conflict of interest.

## ETHICS STATEMENT

The authors have nothing to report.

## Data Availability

Data sets used and/or analysed during the current study are available from the corresponding author upon reasonable request.
